# Opioid Addiction/Pregnancy and Neonatal Abstinence Syndrome (NAS): A Preliminary Open-Label Study of Buprenorphine Maintenance and Drug Use Targeted Psychotherapy (DUST) on Cessation of Addictive Drug Use

**DOI:** 10.3389/fpsyt.2020.563409

**Published:** 2020-09-23

**Authors:** Sarah Tabi, Sarah A. Heitner, Swati Shivale, Scott Minchenberg, Stephen V. Faraone, Brian Johnson

**Affiliations:** ^1^ Department of Psychiatry, State University of New York Upstate Medical University, Syracuse, NY, United States; ^2^ Department of Psychiatry, Perelman School of Medicine, University of Pennsylvania, Philadelphia, PA, United States; ^3^ Department of Psychiatry, Bellevue Hospital Centre and New York University School of Medicine, New York, NY, United States; ^4^ Department of Medicine, Beth Israel Deaconess Hospital and Harvard Medical School, Boston, MA, United States

**Keywords:** neonatal abstinence syndrome, neonatal opioid abstinence syndrome, tobacco and pregnancy, buprenorphine and pregnancy, cold pressor test

## Abstract

**Background:**

Neonatal abstinence syndrome (NAS) is common, expensive, and hurts opioid addicted women and their families. Current treatments do not sufficiently address comorbid addictions, especially tobacco use, among pregnant buprenorphine-maintained women.

**Methods:**

25 consecutive admissions of pregnant, opioid addicted women were treated with buprenorphine maintenance and a novel intervention for pregnant opioid addicted patients, Drug Use Targeted Therapy (DUST). DUST entails a combination of informing women about the impact of various drugs on their fetus, discussing the woman’s thinking about these consequences of drug use, and varying the frequency of psychotherapy; increasing if addictive drugs are used and decreasing if the woman wishes when drug use is stopped.

**Results:**

20/25 remained in treatment until delivery. All 20 women were using addictive drugs at admission. None were planned pregnancies. There was a high prevalence of emotional, physical or sexual abuse, criminal behavior, comorbid psychiatric disorders, and chronic pain. Nineteen stopped all addictive drugs. NAS was present for 5 out of 19 newborns with a duration of hospitalization from 4 to 6 days.

**Conclusions:**

This preliminary open-label case series found that pregnant buprenorphine maintained women can stop tobacco. What has sometimes been termed “neonatal opioid abstinence syndrome” may most accurately be termed, “neonatal opioid/tobacco abstinence syndrome.” If the treatment effectively addresses tobacco use, other addictive drugs are rarely used. DUST resulted in a 95% quit rate for addictive drugs. Pilot data on this new intervention is limited; a case series that does not have a corresponding control group.

## Introduction

Neonatal abstinence syndrome (NAS) is the result of the sudden discontinuation of substances used by the mother during pregnancy. Since 2016, it has also been called, “Neonatal opioid withdrawal syndrome.”(1) The incidence of NAS has quadrupled in 10 years to 6.5 per 1,000 live births and the cost of care has increased by six times in that period to $563 million, based on 2014 statistics (2). NAS causes suffering for neonates. Families must cope with witnessing a multitude of symptoms in the infant, including vomiting, diarrhea, decreased appetite, respiratory dysfunction, nasal congestion, frequent sneezing, crying, tremors, diaphoresis, and spasticity (1). NAS often requires a prolonged hospitalization with an opioid taper (i.e., morphine or methadone) over multiple weeks (1, 3–6). The prevalence of NAS is 55%–94% of newborns whose mothers were addicted to or treated with opioids while pregnant (4). The average duration of hospitalization for neonates is 17 days (4).

The MOTHER study, called “the most rigorous comparison”, (7) showed that pregnant women maintained on buprenorphine were retained at a lower rate, 67% versus methadone, 82%. Hospital stays for neonates lasted 10 days versus 17.5 days for methadone. There was a contingency management component to the study. Subjects were paid for urine drug screens negative for illicit drugs; average $1,500. Heroin use averaged 9 days in the last month, cocaine use 4 days. In addition, 85% of these subjects used tobacco.

The literature on psychotherapy during buprenorphine maintenance was reviewed by Carroll and Weiss in 2017 (8). Four studies that involved cognitive behavioral therapy or counseling had no impact on treatment retention or negative drug screens whereas four contingency management studies had a positive effect on these outcomes.

Tobacco use during pregnancy causes fetal loss, premature rupture of membranes, placental abruption, placenta previa, cleft lip/palate, cardiac, limb and gastrointestinal defects, low birth weight, and newborns with increased respiratory infections, bronchiolitis, otitis media, reactive airway disease, short stature, hyperactivity, obesity, decreased academic performance, and a 34% increase in unexpected infant deaths (7). Tobacco use in pregnant, opioid addicted women is high, between 88% and 95% (9). Tobacco cessation for opioid-maintained women is generally unsuccessful. Our review of the literature, and a 2015 review (10) found three studies. Two of these studies showed that none of these women were able to stop smoking. A 12-week contingency management study resulted in 31% cessation (11). However, it was unclear from the report whether the tobacco abstinence continued until delivery.

What the common comorbid psychopathologies in buprenorphine-maintained pregnant women might be is unexplored. We were unable to find any articles in PubMed regarding this question. One study of opioid use disorder patients receiving opioid substitution treatments showed more severe symptoms in women as measured by both general symptomatic index and symptom checklist 90-revised. Somatization, obsession-compulsion, interpersonal sensitivity, depression, anxiety, phobic anxiety, and paranoid ideation were all more common (12).

We provide preliminary data from a case series employing a unique intervention. Our goal is to provide pilot data preliminary to a future study that incorporates a control group who receive conventional treatment.

## Methods

### Study Design

This is a report of a retrospective chart review of 25 consecutive admissions over nearly four years, 2015–2018.

### Participants

Every pregnant opioid addicted woman who presented for evaluation was taken into treatment. The SUNY Upstate Institutional Review Board (IRB) approved the study protocol. All subjects provided written informed consent prospectively which allowed us to use their de-identified information.

### Baseline Assessment

A holistic evaluation of each pregnant woman resulted in DSM5 diagnoses, a dynamic formulation, and a treatment plan. Multiple pathologies were the rule, not the exception. Each diagnosis resulted in an identified treatment. If the patient had comorbid back pain and Attention Deficit Hyperactivity Disorder (ADHD), these immediately went into the treatment plan.

Every woman had to bring a support person for the initial evaluation. This required asking for help, and creating the start of a recovery environment, before arriving for evaluation. Usually, this support person was the mother of the patient, or the father of the fetus. Consequences of using other drugs were conveyed immediately to the patient and her supporters.

We use the cold pressor test to identify opioid induced hyperalgesia. This intensely experiential demonstration of opioids intensifying pain is shown by plunging the forearm into ice water. We used opponent process theory to explain how opioids intensify pain (13, 14) for our pregnant, opioid using patients with pain complaints. This intervention undercut idealization of opioid use and justification of opioid use for pain.

Psychotherapy was universal. Medications were added for ADHD, depressive disorders and tobacco cessation balancing the ideal of giving no medications if possible against the necessity of pharmacologic treatment for resolution of drug use in the context that abstinence is difficult while comorbid disorders are symptomatic.

### Statistical Analysis

For comparisons between two categorical variables, p-values were generated using the chi-square test. For comparisons between two continuous variables, statistical significance was determined by an unpaired Student’s t-test. Histograms show the mean values with standard deviation. The particular test used for each analysis is indicated in the figure legends. A p-value of < 0.05 was interpreted as statistically significant. All statistical analyses were performed using GraphPad Prism (version 7.0d). 

### Drug Use Targeted Therapy (DUST)

#### Theoretical Basis

DUST is an innovative psychotherapy developed by author Brian Johnson over a period of 5 years, using a theoretical-empirical approach. As we have described elsewhere, the scientific premise of our work, articulated in a theoretical model, posits that opioid use severely compromises the ability to relate because emotional contact is painful while maintained on opioids (15). Our approach took into account that our subjects were likely to experience that emotional contact involved in psychotherapy was aversive. Therefore, a contingency management could be built in, not by providing payment, but rather by allowing our patients to decrease the frequency of psychotherapy as a reward for abstaining from addictive drugs.

#### Therapeutic Alliance

We have not had an evaluation yet of an opioid addicted woman who was not also addicted to tobacco, often with cocaine, amphetamines, alcohol, and marijuana also used. DUST was built around the single treatment goal of a healthy baby—the agreed upon work of the pregnant patient, her supporters, and the treaters. The therapeutic alliance was constructed to include both benign and hostility-containing components. The initial alliance was built around agreement that the welfare of the baby is at risk if the mother continues her addictive drugs.

The emotional intensity of the healthy baby mandate was augmented by a fact sheet on effects of tobacco use on the fetus, read to the support person and treaters by the patient during the evaluation (**Supplementary Table 1**). The goal is to inform, but also to set the stage for psychotherapy done in the play mode, by listing a grim set of harms to the fetus from tobacco but ending with a wrong choice that tobacco use by the mother can also cause alien abduction. Although the topics of psychotherapy can be grim, the overall setting is fun rather than shame or judgment.

Warm and firm limits on drug use facilitate a holding environment. For example, one patient was positive for inhaling tobacco and cocaine on her first psychotherapy hour after intake. She said (jokingly), “What do you expect? I’m a drug addict!” Her baby’s father was in prison and the father’s mother had been the support person. A few weeks later her UDS was negative, she was ebullient, and said how proud the grandmother was about her efforts.

A urine drug screen (and since data collection for this contribution closed an expired carbon monoxide level) is done by the psychotherapist in front of the patient at the start of each DUST encounter. The results set the stage for the subsequent discussion of either abstinence or continued drug use by the patient.

#### Translating Behaviors Into Words

Another psychotherapeutic innovation is DUST’s premise that drug use is “about something.” There is a wild hostility in addictive behaviors (16). This is so apparent to lay observers that some states criminalize addictive drug use by pregnant women and it is considered child abuse in 18 American states (17). We fully subscribe to the hostility of addictive drug use by pregnant women and believe that the problem that allows it to continue is that the intent to harm the fetus is not conscious (18).

Interpretation allows the mother to make a conscious choice about whether to cause harm. With the fact sheet on tobacco use during pregnancy as a beginning, we can ask questions such as, “You know cigarettes are harming your baby, and you are still inhaling them. Tell me about that.” Women were proud of the insights they developed from this investigation. For example, one woman whose mother had been abusive to her recognized that she had been unconsciously replicating that abuse by inhaling tobacco. Cigarette use stopped that moment. If treaters are not judgmental but rather curious, using the therapeutic alliance built in the first encounter and developed over a course of psychotherapy that all parties are deeply invested in a healthy baby as the outcome, women are able to turn malignant behaviors into safe words.

Making tobacco a central focus is not ideological but rather practical. Over the years, we have found that when patients stop tobacco, illicit opioids, and other addictive drugs almost always also stop.

#### Using Countertransference

The addicted person will often attack those who interfere with drug use. If addictive behavior stops, the hostility or other dysphoric experiences enter the relationship with the psychotherapist (19). Therefore, the psychotherapist must be able to tolerate feelings of rage, hate, and hostility both in the patient as they relate to others in their human surround and also as these feelings are stirred up in the countertransference experienced by the psychotherapist. The psychotherapist must master their own aggression and channel it into providing a holding environment that aggressively contains the patient’s rage.

We helped our pregnant women be aware of their hatred and rage. We explained that it’s expression as addictive drug use was hurting their fetuses. We used the power of providing buprenorphine as part of containing this hostility while using our therapeutic alliance as a benevolent force. Women understood the shared goal to have them deliver babies who had to withdraw from buprenorphine only.

#### Combined Psychotherapy and Psychopharmacologic Treatment

In our review of combined psychotherapy/pharmacology treatments in addiction, we found few studies (20). Most treatments use medications alone or psychotherapy alone. DUST is delivered by medical practitioners who concordantly prescribe medications that propitiate abstinence. While buprenorphine maintenance is bedrock, difficulty with tobacco use cessation is targeted with medications such as bupropion and varenicline. DUST is a classic psychotherapy/medication combined treatment. If there is difficulty stopping tobacco, following a risk/benefit discussion about varenicline, the patient was asked to stop tobacco the night before, come to their psychotherapy hour craving nicotine, and take varenicline 0.5 mg in front of the psychotherapist. Nicotine craving disappeared in 20 min. The patient was told that any subsequent nicotine cravings could be responded to with more varenicline. Dosing is variable according to need. Varenicline effect lasts a few hours. The highest dose used in the case series was 3 mg/day taken in 0.5-mg doses. If tobacco is used in place of varenicline, this became a topic for psychotherapy. Other members of the household who are also addicted to tobacco are invited to participate in tobacco cessation treatment.

Because of reports of NAS provoked by the SSRI group of antidepressants or combined opioid/SSRI withdrawal (21) we discontinue SSRIs, evaluate subsequent depressive symptoms, and replace with bupropion if an antidepressant is needed. The general approach was to avoid prescribing anything but buprenorphine. Bupropion or varenicline were given if needed as an adjunct to DUST.

#### Contingency Management

Initial meetings were weekly. Buprenorphine prescriptions were issued for a week. We acknowledged how difficult it is to stop addictive drugs. Our contract was that if the use of all other addictive drugs besides buprenorphine did not end, as verified by urine drug screen and (since data collection ended) expired carbon monoxide after four weeks, we would only prescribe buprenorphine for 3 or 4 days at a time for the next four weeks, with DUST twice a week. If the drug screen was not negative at 8 weeks, DUST was daily 5 days per week with only 1 day of buprenorphine prescribed at a time.

When it proved difficult to stop drug use the attending psychiatrist provided his cell phone number and asked that the patient call BEFORE any more drug use. This did not cause any undue burden on the psychiatrist. The reason for this is that patients tended not to call, but to come back not using addictive drugs, as if they thought of calling and then did not use the drug.

Once the drug screen turned negative for everything except buprenorphine, we were in a continuation phase of treatment, the acute interventions were over, and visits became monthly. The only cotinine positive UDS we have found at this point have to do with second hand smoke exposure. The mother becomes aware of the hostility of her loved ones, furthering discussions of safety and health. Weekly psychotherapy continuation was offered, but nearly universally declined. Our understanding is that buprenorphine maintenance makes emotional contact painful (22). We take advantage of this phenomenon to make DUST a form of contingency management. The reward of decreasing painful emotional contact motivates the patient to remain abstinent.

*Summary: Contingency management involves being allowed to come to treatment less often when UDS shows abstinence from addictive drugs.*

**Table:** 

Visit	UDS Result	Treatment Response
1	Pos	Come back in a week
	Neg	Come back in a month
2–4	Pos	Come back in a week
	Neg	Come back in a month
5–8	Pos	Come back in 3–4 days
	Neg	Come back in a month
9–Delivery	Pos	Come back 5 days/week
	Neg	Come back in a month

## Results

### Patient Characteristics

The average (mean) age of the patients was 29 (range 20–36). Ten women were in the first trimester at intake, 10 in the second, 5 in the third. None of the pregnancies were planned, but all were desired once known. There are 14/25 women who gave a history of physical, emotional, or sexual abuse, and 10/25 had a history of criminal conviction. The cold pressor time was tested for 10/25. It averaged 23 s (range 0–72) in the context that 23 female controls on our service, average age 52, averaged a cold pressor time of 111 s (20). The average score on the faces pain scale for 23 women was 3.4 (range 0–9). In addition, 25/25 women used tobacco. Other substances used at intake were cannabis 6, cocaine 4, benzodiazepines 4, methamphetamine 2, alcohol 1.

Comorbid psychiatric disorders were: attention deficit hyperactivity disorder 10, borderline personality 9, major depressive disorder 3, persistent depressive disorder 3, anorexia nervosa 1. Most women were poor, disadvantaged and functioned at a low level psychologically. A total of 7 women were prescribed bupropion, 300 or 450 mg/day. Five women who could not stop tobacco without medication or with only bupropion were given varenicline.

### Treatment Outcomes

One patient had a spontaneous miscarriage at 5 months gestation and was removed from data analysis. Four discontinued treatment. Of the remaining 20 patients all stopped tobacco. One patient insisted she needed sertraline and clonazepam. Her child had significant NAS. There are 19/20 women who stopped all addictive drugs, 13/20 women were consistently negative for cotinine, and 7/20 were positive for cotinine but avidly insisted that they were no longer inhaling tobacco. What we have found since we began using a carbon monoxide meter is that women who are exposed to second hand smoke that have cotinine positive urines. They made comments such as, “My boyfriend smoked in the truck while he drove me to this appointment.” At the time of delivery, women who were abstinent from addictive drugs were also in remission from depressive disorders.

**Figure 1:**
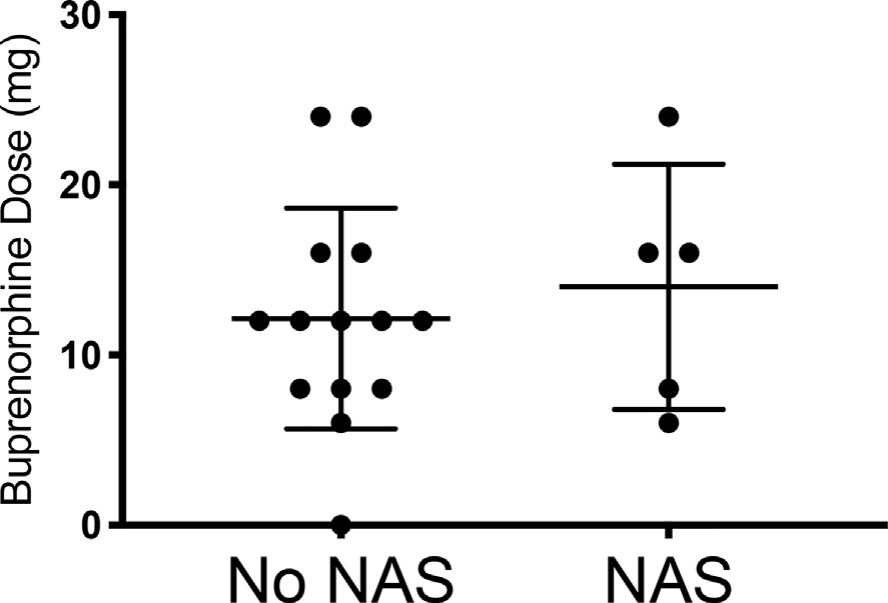
Comparing the initial buprenorphine doses between patients whose infants had NAS v those who did not develop NAS. No NAS mean = 12.14 [Stdev 6.49] n=14; NAS mean 14.00 [Stdev 7.21] n=5; Two- tailed t-test [p=0.60].

**Figure 2:**
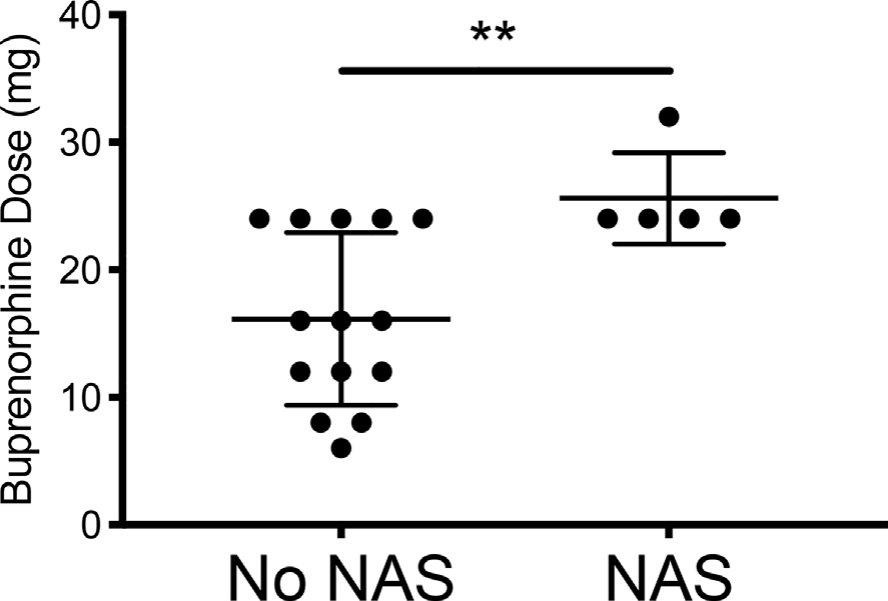
Comparing the final buprenorphine doses between patients whose infants had NAS v those who did not develop NAS. No NAS mean = 16.14 [Stdev 6.77] n=14; NAS mean 25.60 [Stdev 3.58] n=5; Two- tailed t-test [**p=0.009].

While initial buprenorphine dose was indistinguishable between mothers whose children did and did not develop NAS, the minor NAS observed in the five women whose children did develop transient NAS was significantly related to dose escalation of buprenorphine (**Figures 1**, **2** and **Table 1**).

**Table 1:** Escalation of buprenorphine dose during treatment was significantly linked with NAS.

	NAS n = 5	No NAS n = 14	p-value	Test
Dose Escalation	4	6	0.15	Chi-Square
Initial Buprenorphine Dose	14.00 ± 7.21	12.14 ± 6.49	0.60	Unpaired t-test
**Final Buprenorphine Dose**	**25.60 ± 3.58**	**16.14 ± 6.77**	**0.009**	** Unpaired t-test**

The significance of the bolded data is p = 0.009.

## Discussion

Our main findings are:

1. DUST results in a high quit rate for addictive drugs. The high rate of quitting seems related to a sustained engagement in psychotherapy that takes into account the limitation of relatedness seen during opioid maintenance, beginning the treatment with clear education, a handout on tobacco shared with an important supporter, and then investigation of impediments to abstinence. Asking about the patient’s thinking regarding injuring their growing fetus with drug use was regarded as a non-judgmental statement of fact supported by scientific evidence. The DUST intervention made conscious the hostility involved in drug use while pregnant and often helped transfer the hostility from the fetus toward the therapist. The therapist was then able to contain the aggression resulting in clinical improvement. The therapeutic alliance with play as the modality of engagement made it clear that the enemy was not the pregnant patient, but rather the entities who sell drugs.
2. What has sometimes been termed “neonatal opioid abstinence syndrome” may be combined drug withdrawal. It might most accurately be termed, “neonatal opioid/tobacco abstinence syndrome” because tobacco seems to be a significant factor in NAS intensity and duration. Tobacco seems to be the most common drug in terms of multiple drug use. If treatment brings tobacco use into remission other drugs are rarely used.
3. Contingency management is built in, not with financial payments, but by understanding that opioid maintenance makes human contact uncomfortable, informing treaters that the “reward” for abstinence can be allowing disengagement. This makes contingency management more widely available as a therapeutic technique. Like many other treatment programs that barely make our expenses, we would find it difficult to have financial payments as part of the care.
4. Part of the empathy involved from the treater side was that being engaged emotionally was tolerated by the patients because of their commitment to having a healthy baby. We appreciated that there were many difficulties involved in an ongoing treatment. Giving up drugs is hard, especially when pregnant!
5. Intimate partner violence is an important aspect to consider in terms of treatment (6). We extend the concept of intimate partner violence toward mother and fetus to include second hand smoke. Again, this is not a conscious hostility. A negative exhaled carbon monoxide combined with a positive urine cotinine allows the pregnant patient to be more aware and to warn their emotional supporters, that environmental nicotine is getting into mother and fetus. Finding positive urine cotinine after inhaling cigarettes has stopped facilitates the pregnant woman to ask supporters to care for her and the fetus by avoiding smoking near her.
6. In contrast with an authoritative review that explained buprenorphine dose was not related to NAS incidence (6). when other drugs are removed, buprenorphine dose was correlated with incidence of NAS.

There are limitations to this study:

1. This was an exploratory case series developed on an Addiction Medicine service. It is only a preliminary report.
2. Results were collected as part of routine treatment rather than assessed by a blinded third party.
3. The case series is small.
4. There was no comparison treatment group.
5. Expired carbon monoxide was not measured. Cotinine positive urines obtained while the mother unequivocally stated that she had stopped nicotine use were counted as quits. Introduction of carbon monoxide meter after this case series closed revealed that persistent cotinine positive urine drug screens was probably caused by second hand smoke. However, it is possible that the self-report may have been dishonest.
6. Longer term outcomes of maternal health such as postnatal persistence of addictive drug use, overall health and competence to parent, as well as newborn physical health, appropriate developmental milestones, growth, and bonding between mother and infant, were not undertaken.
7. Generalizability of results to other treatment services cannot be addressed with our data.
8. Our literature review revealed that comparisons of our outcomes to other treatments is difficult because:
  - There do not seem to be any reports of successfully helping pregnant buprenorphine-maintained women abstain from tobacco and other addictive drugs and,
  - We could find no reports of treatment of comorbid psychiatric disorders in pregnant buprenorphine-maintained women.

## Conclusion

We have created a novel intervention for pregnant opioid addicted patients on buprenorphine maintenance that is promising regarding cessation of addictive drugs and that may decrease the prevalence and severity of NAS. Compared to published outcome studies:

1. We are using a version of contingency management that does not require cash payments. The reward is distance from treaters. It makes the DUST approach applicable to treatment facilities such as ours that treat mostly poor patients and operate on a budget that would not allow payments that are commonly as high as $1,500 for negative urine drug screens (1).
2. DUST takes advantage of all available aspects of treatment; consideration by pregnant women about what drugs do to their fetuses in a supportive and non-judgmental environment without need for the therapeutic relational bond that dooms psychotherapy on buprenorphine-maintenance, (8) requiring patients to ask for help before they arrive, facilitating support for abstinence, and having a healthy baby, and medications such as bupropion and varenicline if needed, to help patients abstain from the decisive addictive drug, tobacco.
3. Even in the best available study, MOTHER, (1) retention on buprenorphine was 2/3, heroin use averaged 9 days in the last month, cocaine use 4 days and 85% of subjects used tobacco. Our very preliminary pilot study resulted in an unusually high retention rate, 80%, and abstinence for 95% of women followed through delivery.
4. NAS was reported by 5/19 of our abstinent subjects as present but a transient, less than a day, experience of newborns. This is far below the 55%–94% in other studies (4). None of the newborns was treated with opioid medications versus the vast majority reported in other studies.

## Data Availability Statement

The datasets presented in this article are not readily available because: Deidentified information was used but is not to be disseminated. Requests to access the datasets should be directed to johnsonb@upstate.edu.

## Ethics Statement

The studies involving human participants were reviewed and approved by SUNY Upstate Institutional Review Board. The patients/participants provided their written informed consent to participate in this study.

## Author Contributions

ST and SH wrote the first draft. BJ’s service is where the case series took place. He invented DUST. SS treated the first patients in the case series and contributed to writing. SM did the statistical analysis with input from SF, who also extensively wrote the manuscript.

## Conflict of Interest

The authors declare that the research was conducted in the absence of any commercial or financial relationships that could be construed as a potential conflict of interest.
