# Supplementary material for: Opioid Addiction/Pregnancy and Neonatal Abstinence Syndrome (NAS): A Preliminary Open-Label Study of Buprenorphine Maintenance and Drug Use Targeted Psychotherapy (DUST) on Cessation of Addictive Drug Use
Source: Front Psychiatry. 2020 Sep 23;11:563409. doi: 10.3389/fpsyt.2020.563409 (PMC7538830; doi:10.3389/fpsyt.2020.563409)
Supplement: Supplementary file 1 [file DataSheet_1.pdf]

Test: Pregnancy and Cigarettes

Which of these is NOT an effect of smoking cigarettes during pregnancy?

Only ONE of these is not a risk caused by smoking during pregnancy.

1. Permanent loss of intelligence of your child, average 7 IQ points
2. Three times greater risk of schizophrenia caused by fetal and childhood exposure
3. Greater risk of Attention Deficit Hyperactivity Syndrome
4. Placenta previa (The placenta covers the cervix, leading to dangerous bleeding.)
5. Placental abruption (The placenta separates prematurely, endangering both mother and baby.)
6. Thyroid dysfunction
7. Ectopic pregnancy
8. Premature rupture of membranes (The sac the baby lives in is punctured, risking delivery before the lungs are mature.)
9. Stillbirth
10. Intrauterine growth restriction caused by starving the growing fetus of oxygen, average loss of weight at birth at least ½ pound
11. 2-3 times greater risk of Sudden Infant Death Syndrome (SIDS) which is the death of a baby with no cause after birth
12. Asthma after birth
13. Greater risk of congenital heart defects
14. Greater risk of alien abduction immediately after birth
